# nestedcv: an R package for fast implementation of nested cross-validation with embedded feature selection designed for transcriptomics and high-dimensional data

**DOI:** 10.1093/bioadv/vbad048

**Published:** 2023-04-13

**Authors:** Myles J Lewis, Athina Spiliopoulou, Katriona Goldmann, Costantino Pitzalis, Paul McKeigue, Michael R Barnes

**Affiliations:** Centre for Experimental Medicine & Rheumatology, William Harvey Research Institute, Barts and The London School of Medicine and Dentistry, Queen Mary University of London, London EC1M 6BQ, UK; Alan Turing Institute, London NW1 2AJ, UK; Usher Institute, College of Medicine and Veterinary Medicine, University of Edinburgh, Edinburgh EH16 4UX, UK; Centre for Experimental Medicine & Rheumatology, William Harvey Research Institute, Barts and The London School of Medicine and Dentistry, Queen Mary University of London, London EC1M 6BQ, UK; Centre for Translational Bioinformatics, William Harvey Research Institute, Barts and The London School of Medicine and Dentistry, Queen Mary University of London, London EC1M 6BQ, UK; Centre for Experimental Medicine & Rheumatology, William Harvey Research Institute, Barts and The London School of Medicine and Dentistry, Queen Mary University of London, London EC1M 6BQ, UK; Usher Institute, College of Medicine and Veterinary Medicine, University of Edinburgh, Edinburgh EH16 4UX, UK; Alan Turing Institute, London NW1 2AJ, UK; Centre for Translational Bioinformatics, William Harvey Research Institute, Barts and The London School of Medicine and Dentistry, Queen Mary University of London, London EC1M 6BQ, UK

## Abstract

**Motivation:**

Although machine learning models are commonly used in medical research, many analyses implement a simple partition into training data and hold-out test data, with cross-validation (CV) for tuning of model hyperparameters. Nested CV with embedded feature selection is especially suited to biomedical data where the sample size is frequently limited, but the number of predictors may be significantly larger (*P* ≫ *n*).

**Results:**

The *nestedcv* R package implements fully nested *k *×* l*-fold CV for lasso and elastic-net regularized linear models via the *glmnet* package and supports a large array of other machine learning models via the caret framework. Inner CV is used to tune models and outer CV is used to determine model performance without bias. Fast filter functions for feature selection are provided and the package ensures that filters are nested within the outer CV loop to avoid information leakage from performance test sets. Measurement of performance by outer CV is also used to implement Bayesian linear and logistic regression models using the horseshoe prior over parameters to encourage a sparse model and determine unbiased model accuracy.

**Availability and implementation:**

The R package *nestedcv* is available from CRAN: https://CRAN.R-project.org/package=nestedcv.

## 1 Introduction

The motivation for this package is to provide functions which help with the development and tuning of machine learning models in biomedical data where the sample size is frequently limited, but the number of predictors may be significantly larger (*P* ≫ *n*). While most machine learning pipelines involve splitting data into training and testing cohorts, typically 2/3 and 1/3, respectively, medical datasets may be too small for this, and so determination of accuracy in the left-out test set suffers because the test set is small. Nested cross-validation (CV) (Stone, 1977) provides a way to get round this, by maximizing use of the whole dataset for testing overall accuracy, while maintaining the split between training and testing.

In addition, typical biomedical datasets often have many 10 000s of possible predictors, so filtering of predictors is commonly needed. However, it has been demonstrated that filtering on the whole dataset creates a bias when determining accuracy of models (Vabalas *et al.*, 2019). Feature selection of predictors should be considered an integral part of a model, with feature selection performed only on training data. Then the selected features and accompanying model can be tested on hold-out test data without bias. Thus, any filtering of predictors is performed within the CV loops, to prevent test data information leakage.

## 2 Description

### 2.1 Nested cross-validation

This package enables nested CV to be performed using the commonly used *glmnet* package, which fits elastic net regression models (Zou and Hastie, 2005), and the caret package (Kuhn, 2008), which is a general framework for fitting a large number of machine learning models. In addition, *nestedcv* adds functionality to enable CV of the elastic net alpha parameter when fitting *glmnet* models.

*nestedcv* partitions the dataset into outer and inner folds (default 10 × 10 folds). The inner fold CV, (default is 10-fold), is used to tune optimal hyperparameters for models. Then the model is fitted on the whole outer training fold and tested on the left-out data from the outer fold. This is repeated across all outer folds (default 10 outer folds), and the pooled unseen test predictions from the outer folds are compared against the true results for the outer test folds and the predicted probabilities concatenated, to give measures of accuracy [e.g. ROC AUC (receiver operating characteristic curve area under curve) and accuracy for classification, or RMSE (root mean square error) for regression] across the whole dataset. A final round of CV is performed on the whole dataset to determine hyperparameters to fit the final model to the whole data, which can be used for prediction with external data. For speed, parallelization of the outer CV loops has been incorporated, since some feature selection methods can also be time consuming. Parallelization uses parallel::mclapply to allow forking where available (non-windows systems) for efficient memory usage.

### 2.2 Variable selection

While some models such as glmnet allow for sparsity and have variable selection built-in, many models fail to fit when given massive numbers of predictors, or perform poorly due to overfitting without variable selection. In addition, in medicine one of the goals of predictive modelling is commonly the development of diagnostic or biomarker tests, for which reducing the number of predictors is typically a practical necessity. A collection of filter functions for feature selection are provided, including simple, extremely fast univariate filters such as t-test, Wilcoxon test, one-way ANOVA and Pearson/Spearman correlation, as well as more complex filters such as random forest variable importance, ReliefF (Kononenko *et al.*, 1997) from the *CORElearn* package and *Boruta* (Kursa and Rudnicki, 2010). Filters are designed to be embedded within the outer loop of the nested CV and custom-made filters are supported. A comparison of feature selection methods applied to gene expression datasets showed that a simple *t*-test often performed best in terms of predictive performance and stability (Haury *et al.*, 2011).

### 2.3 Imbalanced datasets

Class imbalance is known to impact on model fitting for certain model types, e.g. random forest. Models may tend to aim to predict the majority class and ignore the minority class as selecting the majority class can give high accuracy purely by chance. While performance measures such as balanced accuracy can give improved estimates of model performance, techniques for rebalancing data have been developed. These include: random oversampling of the minority class; random undersampling of the majority class; combination of oversampling and undersampling; synthesizing new data in the minority class, e.g. SMOTE (synthetic minority over-sampling technique) (Chawla *et al.*, 2002). These are available within nestedcv using the balance argument to specify a balancing function. Balancing can have a deleterious effect on regression (van den Goorbergh *et al.*, 2022), but is known to benefit some tree-based models such as random forest (Chen *et al.*, 2004).

### 2.4 Importance of nested CV

**Fig. 1.**
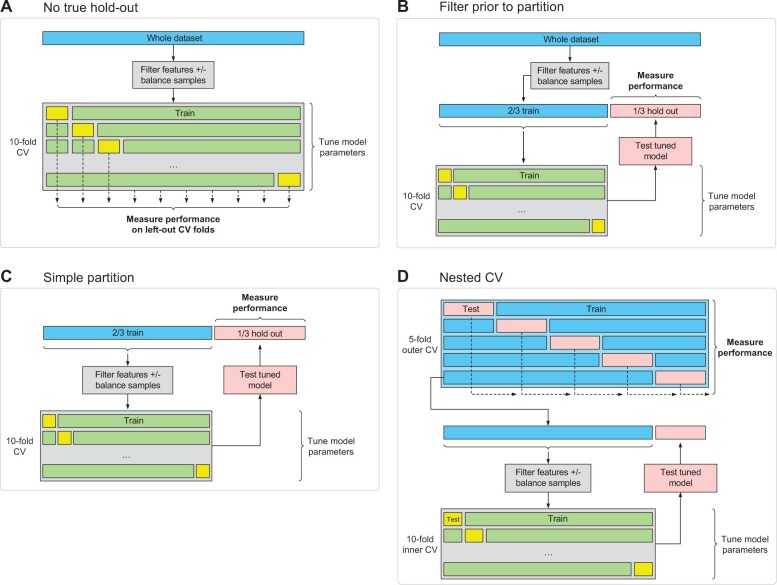
Schemes for fitting machine learning models with CV. (**A**, **B**) Commonly implemented schemes which lead to biased measures of performance due to feature selection being applied to the whole dataset. (**C**, **D**) Valid schemes which include feature selection, model fitting and unbiased measurement of performance using either (C) a simple partition or (D) nested CV

Figure 1A and B shows two commonly used, but biased methods in which CV is used to fit models, but the result is a biased estimate of model performance. In scheme A, there is no hold-out test set at all, so there are two sources of bias/data leakage: first, the filtering on the whole dataset, and second, the use of left-out CV folds for measuring performance. Left-out CV folds are known to lead to biased estimates of performance as the tuning parameters are ‘learnt’ from optimizing the result on the left-out CV fold. In scheme B, the CV is used to tune parameters and a hold-out set is used to measure performance, but information leakage occurs when filtering is applied to the whole dataset. Unfortunately, this practice is commonly observed in many studies which apply differential expression analysis on the whole dataset to select predictors which are then passed to machine learning algorithms. Figure 1C and D shows two valid methods for fitting a model with CV for tuning parameters as well as unbiased estimates of model performance. Figure 1C is a traditional hold-out test set, with the dataset partitioned 2/3 training, 1/3 test. Notably the critical difference between scheme B above, is that the filtering is only done on the training set and not on the whole dataset. Figure 1D shows the scheme for fully nested CV. Note that filtering is applied to each outer CV training fold. The key advantage of nested CV is that outer CV test folds are collated to give an improved estimate of performance compared to scheme C since the numbers for total testing are larger.

Similarly, in *nestedcv* balancing is performed only on the outer training folds, immediately prior to filtering of features. This is important as balancing the whole dataset outside the outer CV would lead to data leakage of outer CV hold-out samples into the outer training folds, leading to performance bias.

Alternative methods for producing less biased estimates of model performance have been proposed including Bootstrap Bias Corrected CV (BBC-CV) (Tsamardinos *et al.*, 2018). This is based on harnessing out-of-sample predictions generated during standard CV for tuning hyperparameters. However, this method does not easily incorporate feature selection/filtering as an added step while measuring model performance, and to our knowledge the only implementation available for the purpose of comparison is written in MATLAB. Since the bootstrapping step needs to be performed 100s (if not 1000s) of times to correctly estimate the bias in performance from standard CV, this method may not be much faster than nested CV, especially with the advent of easier deployment of parallel processing.

### 2.5 Fitting the final model

Figure 1D shows the scheme for nested CV to measure performance of the selected model. The final model is determined by following the same steps as are applied to the outer training folds, but this time to the whole dataset to derive the final fitted model. Namely these steps are:

1. Filter predictors based on the whole data.
2. Optionally apply balancing functions to the samples (e.g. random over/under sampling or SMOTE).
3. Split into CV folds, use (10×) CV to tune hyperparameters of the final model.
4. Fit final tuned model to whole data; return this model.

## 3 Implementation

### 3.1 Models which require parameter tuning

**Fig. 2.**
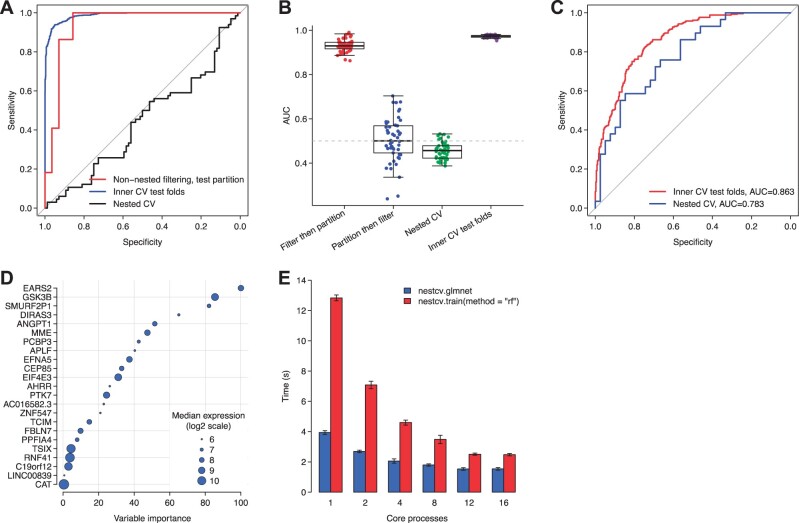
Performance of nested CV in simulated and real-world examples. (**A**) Using a simulated dataset of 50 000 predictors generated from gaussian noise, filtering of predictors on the whole dataset and measurement of performance using train/test partition (“non-nested filtering, test partition”) leads to biased estimates of performance as shown by receiver operating characteristic (ROC) curve plots. Use of predictions from CV test folds also shows performance bias. Fully nested CV shows the true predictive performance is poor. (**B**) Same simulation as in (A) performed 50× showing that filtering on the whole dataset followed by test of performance by simple train-test partition (‘Filter then partition’) shows biased performance in ROC area under curve (AUC). Simple partition followed by filtering on the training data (‘Partition then filter’) and fully nested CV are unbiased in that they show that the true predictive performance of the dataset is poor. However, nested CV shows lower variance across repeats. (**C**) In a real-world example from the R4RA clinical trial, RNA-Seq gene expression from synovial biopsies was trained with an elastic-net predictive model to predict clinical response to the drug rituximab using nested CV to measure performance by ROC area under curve (AUC). (**D**) Bubble plot showing genes from the final fitted model in (C) ranked by variable importance and with diameter showing median level of gene expression. (**E**) Benchmark of 20 runs of fitting either glmnet or random forest 10 × 10-fold nested CV models to the real-world data shown in (C) and (D), showing mean ± SD performance improvement with parallel processing on an 8-core Intel Core i9 processor

The following simulated example demonstrates the bias intrinsic to datasets where *P* ≫ *n* when applying filtering of predictors to the whole dataset rather than to training folds. In this example the dataset is pure gaussian noise (code is included in the *nestedcv* R package vignette). In the first scenario, predictors are filtered on the whole dataset to select the top 100 predictors based on simple *t*-test. The data is partitioned 2/3 train, 1/3 test and an elastic net model is trained to the training data and tested on the test data. In this situation (equivalent to Fig. 1B) filtering of predictors on the whole dataset is a source of leakage of information about the test set, leading to substantially overoptimistic performance on the test set as measured by ROC AUC (Fig. 2A, red line). In comparison nested CV (Fig. 2A, black line) correctly reports an AUC close to 0.50 showing that the dataset lacks predictive attributes. Of note, the inner CV test predictions from nested CV still show a performance bias (Fig. 2A, blue line) since the *glmnet* hyperparameters across folds are chosen based on highest performance. The simulation was performed 50 times (Fig. 2B) with addition of comparing nested CV against simple 2:1 train/test partition where filtering has been performed only on the 2/3 training dataset akin to the scheme shown in Figure 1C. This shows that simple train-test partition is also unbiased, but shows greater variance in performance estimate compared to nested CV.

In a real-world example, RNA-Sequencing gene expression data from synovial biopsies from patients with rheumatoid arthritis in the R4RA randomized clinical trial (Humby et al., 2021; Rivellese *et al.*, 2022) is used to predict clinical response to the biologic drug rituximab. Treatment response is determined by a clinical measure, namely Clinical Disease Activity Index (CDAI) 50% response, which has a binary outcome: treatment success or failure (response or non-response). This dataset contains gene expression on over 50 000 genes in arthritic synovial tissue from 133 individuals, who were randomized to two drugs (rituximab and tocilizumab). First, we remove genes of low expression using a median cut-off (this still leaves >16 000 genes), and we subset the dataset to the rituximab treated individuals (*n* = 68). Nested CV using a univariate filter and a glmnet model reaches an AUC of 0.783 (Fig. 2C). *nestedcv* will also provide the left-out test folds from the inner CV for measurement of performance, but as shown in Figure 2C this AUC of 0.863 is inflated in comparison to the true nested CV performance result. Following model parameter tuning by inner CV, *nestedcv* automatically fits the final model to the whole dataset, which in this case has 22 genes shown in order of variable importance with the level of expression overlaid to highlight the most important and highly expressed genes (Fig. 2D).

Benchmarking of this real-world application of *nestedcv* to the R4RA dataset was performed on an Intel Core i9 processor (system Mac OS, Rstudio environment) with 8 physical cores and 16 virtual cores by hyperthreading to compare speed up from parallelization against single core performance (Fig. 2E). Benchmark of 20 runs showed that parallelization by setting the cv.cores argument improved single core performance by up to 5.0 times for random forest model fitted using nestcv.train() which applies nested CV to caret models. Improvement for nestcv.glmnet() was less (up to 2.5 times) since the original *glmnet* code is already substantially optimized in C++. By default, parallelization is applied to the outer CV loop only, to avoid recursive/nested parallelization of inner CV folds which could lead to excessive numbers of processes being spawned. We generally find that speed up tends to plateau once the number of processes reaches the number of physical cores, since all cores become saturated and there are both time and memory overheads for spawning additional processes.

### 3.2 Models which do not require parameter tuning

nestedcv also includes a function outercv which allows for measurement of model performance in small datasets without the inner CV loop for parameter tuning. Importantly feature filtering is nested within the outer CV loop. This is only suitable for models that do not require tuning of parameters against performance on test data. Such models include Bayesian shrinkage models where the shrinkage parameters can be learned from the data as part of the model-fitting routine. It also includes models where the parameters are fixed, such as random forest models where the number of trees has been shown not to require tuning (Probst and Boulesteix, 2018). The justification is that in some models unnested feature selection may be a more important source of performance metric bias than model tuning (Vabalas *et al.*, 2019). For Bayesian shrinkage models, *nestedcv* provides the function model.hsstan which can be used with outercv for fitting Bayesian linear and logistic regression models using the horseshoe prior over parameters to encourage a sparse model (Piironen and Vehtari, 2017). Models are fitted using the *hsstan* R package, which performs full Bayesian inference through a Stan implementation (Carpenter *et al.*, 2017). In Bayesian inference model meta-parameters such as the amount of shrinkage are also given prior distributions and are thus directly learned from the data through sampling. This bypasses the need to cross-validate results over a grid of values for the meta-parameters, as would be required to find the optimal lambda in a lasso or elastic-net model.

## 4 Summary

In summary, the *nestedcv* package implements fully *k *×* l*-fold nested CV while incorporating feature selection algorithms within the outer CV loops. It adds the capability of nested CV to the caret machine learning framework in widespread use. nestedcv is designed to help measure the performance and stability of predictive models in biomedical datasets with small sample size but large numbers of parameters (*P* ≫ *n*). The package is user-friendly, fast and convenient. It automatically collates the outer fold test results to give performance metrics as well as fitting a final model on the whole dataset. Detailed information and examples of usage are included in the vignette hosted alongside the package on CRAN.

## Data Availability

The data underlying this article are available in ArrayExpress accession ID E-MTAB-11611 and can be downloaded from https://www.ebi.ac.uk/arrayexpress/experiments/E-MTAB-11611. All code used in this article is available through CRAN and GitHub from https://github.com/myles-lewis/nestedcv.
